# Synthesis of Analcime and ZSM-5 Zeolite by Diatomite Without Organic Structure-Directing Agent and Adsorption Properties of Their Acid-Modified Samples on Toluene

**DOI:** 10.3390/nano16140863

**Published:** 2026-07-13

**Authors:** Fanghui Pan, Jianxiang Wang, Javed Iqbal, Fei Yu, Jie Ma

**Affiliations:** 1 Water Resources and Water Environment Engineering Technology Center, Xinjiang Key Laboratory of Engineering Materials and Structural Safety, School of Civil Engineering, Kashi University, Kashi 844000, China; jma@tongji.edu.cn; 2 Research Center for Environmental Functional Materials, State Key Laboratory of Water Pollution Control and Green Resource Recycling, College of Environmental Science and Engineering, Tongji University, Shanghai 200092, China; 2130553@tongji.edu.cn; 3 Department of Chemistry, College of Science, University of Bahrain, Zallaq 1054, Bahrain; javed.iqbal@uaf.edu.pk; 4 College of Oceanography and Ecological Science, Shanghai Ocean University, No 999, Huchenghuan Road, Shanghai 201306, China; 5 Shanghai Institute of Pollution Control and Ecological Security, Shanghai 200092, China

**Keywords:** volatile organic compounds, toluene adsorption, acid modification, seed-induced crystallization, pore diffusion

## Abstract

Zeolites are porous aluminosilicate crystalline materials that are widely used for the adsorption of volatile organic compounds (VOCs). The synthesis of zeolites without organic structure-directing agents (OSDAs) is attractive because of its low cost and environmental friendliness. In this study, analcime and the ZSM-5 zeolite were synthesized from natural diatomite under OSDA-free conditions through different crystallization routes. Analcime was prepared by regulating the hydrothermal conditions, while the ZSM-5 zeolite was synthesized by combining hydrothermal condition regulation with seed-induced crystallization. Hydrochloric acid modification was further used to improve the pore structures and adsorption properties of the zeolites. The optimum acid treatment conditions were 1.0 mol·L^−1^ HCl for analcime and 0.5 mol·L^−1^ HCl for the ZSM-5 zeolite. After acid modification, the specific surface area and pore volume of analcime increased to 271.7 m^2^·g^−1^ and 0.130 cm^3^·g^−1^, respectively, and its tolune adsorption capacity increased from 18.3 mg·g^−1^ to 23.2 mg·g^−1^, corresponding to a 26.6% improvement. For the ZSM-5 zeolite, the optimal modified sample showed a specific surface area of 307.9 m^2^·g^−1^, a pore volume of 0.172 cm^3^·g^−1^, and a toluene adsorption capacity of 65.4 mg·g^−1^, which was 5.5% higher than that of the unmodified sample. Adsorption kinetic analysis indicated that pore diffusion played an important role in toluene adsorption, while acid modification introduced additional acid sites that contributed to chemisorption. Overall, the ZSM-5 zeolite showed a higher adsorption capacity than analcime because of its larger surface area, higher pore volume, and more accessible adsorption sites. This study provides a low-cost and environmentally friendly route for preparing diatomite-derived zeolite adsorbents for VOC removal.

## 1. Introduction

Volatile organic compounds (VOCs) are organic compounds that can escape into the air as gaseous molecules and have a saturated vapor pressure higher than 133.32 Pa at room temperature [1,2,3]. VOCs are often toxic, hazardous, and harmful to the environment [4]. The effective treatment of VOC pollutants is an important issue in environmental protection. Conventional VOC treatment technologies include thermal incineration, catalytic oxidation, adsorption, etc. [5,6,7]. Incineration consumes excessive energy and is prone to produce harmful by-products. Catalytic oxidation has been developed in recent years to achieve low-temperature, flameless combustion at 200~400 °C with the assistance of catalysts. However, the method is more suitable for treating high VOC concentrations above 5000 ppm. Adsorption can enrich VOC molecules on high-performance adsorbents by transferring them from the gas phase to the solid phase, which is beneficial for subsequent treatment. After a long period of development, adsorption has become a mature, simple, and practical VOC removal technology. As pollutant types continue to increase and many emerging treatment technologies remain immature, adsorption still plays an important role in VOC removal.

Porous materials that can be used as sorbents for VOCs include activated carbon, metal–organic frameworks (MOFs), zeolite molecular sieves, etc. [8,9,10]. Among these materials, zeolite molecular sieves are crystalline aluminosilicates with highly stable frameworks. Their ordered micropores can be tuned at the molecular scale, making them suitable for VOC adsorption [11]. Analcime and ZSM-5 were selected because they represent two different zeolite frameworks that can be synthesized from silica-rich diatomite under OSDA-free hydrothermal conditions. Analcime has a high framework Al content and a relatively simple synthesis route, making it suitable for studying acid-induced dealumination and pore formation. ZSM-5 has an MFI framework with intersecting microporous channels, high stability, and proven potential for VOC adsorption. A comparison of these two diatomite-derived zeolites allows us to evaluate how framework structure and acid modification affect toluene adsorption performance. Natural minerals generally contain silicon and are widely distributed and accessible, which can theoretically be used as raw materials for zeolite synthesis [12,13,14,15]. Yue et al. used activated diatomite and regolith as raw materials and utilized the crystallization-inducing effect of tetraethylammonium hydroxide (TEAOH) to successfully synthesize β-zeolites with pure crystalline phases [16]. You et al. synthesized beta zeolite, ZSM-5 zeolite, and SSZ-13 zeolite using different organic structure-guiding agents (OSDAs) with cheap kaolin as the main raw material [17]. The synthesized zeolites had good crystalline phase, high specific surface area, and could be used as sorbent for VOCs. However, the OSDAs used in these studies are expensive, and their removal by calcination may produce hazardous substances.

Therefore, the OSDA-free synthesis of zeolites is an important research topic. Since long-chain organic compounds are not used, OSDA-free synthesis can avoid secondary pollution during calcination and reduce synthesis costs [18,19,20]. The main challenge in OSDA-free zeolite synthesis is the lack of structure-directing groups to induce zeolite crystallization. Modulation is needed to obtain a suitable hydrothermal crystallization environment or to induce crystallization using crystal seeds. Adamu et al. constructed a suitable environment for the crystallization of mordenite (MOR) by modulating the synthesis conditions and synthesized pure mordenite under OSDA-free and crystal seed-free conditions [21]. Yao et al. constructed a suitable environment for the crystallization of X zeolite under OSDA-free conditions and successfully synthesized X zeolite, verifying the feasibility of OSDA-free zeolite synthesis from diatomite [22]. Similarly, Y zeolites and LTA zeolites can be synthesized by constructing suitable hydrothermal crystallization environments [23,24,25]. On the basis of regulating a reasonable crystallization environment, the use of crystal seeds with similar structural units can induce and promote the crystallization of zeolites of the same type [26,27,28]. Liu et al. successfully induced the generation of a ZSM-5 zeolite with the same MFI-type structure using crystal seeds with MFI-type topology [29]. Yue et al. synthesized the ZSM-5 zeolite with the assistance of ZSM-5 crystal seeds using diatomite and regolith as the main raw materials, showing that the crystal seeds can also induce crystalline zeolite from natural mineral raw materials [30].

Although OSDA-free zeolite synthesis from natural minerals has been reported, most previous studies focused on the synthesis of a single zeolite phase or on optimizing crystallization conditions. Less attention has been paid to the controllable preparation of different zeolite adsorbents from the same natural mineral source and to the relationship between acid-induced pore regulation and VOC adsorption performance. In particular, a direct comparison between the diatomite-derived analcime and ZSM-5 zeolite, including their structural evolution after acid modification and their adsorption behavior toward toluene, has rarely been reported.

In this work, analcime was synthesized from diatomite by controlling the hydrothermal conditions under OSDA-free environment. The ZSM-5 zeolite was synthesized by combining hydrothermal condition regulation with seed-induced crystallization. Hydrochloric acid modification was used to optimize the pore structures of the two zeolites. The crystal phase, morphology, specific surface area, pore size, and acid site properties of the samples were characterized by XRD, scanning electron microscopy (SEM), X-ray fluorescence (XRF), N_2_ adsorption–desorption, Fourier transform infrared spectroscopy (FTIR), and NH_3_ temperature-programmed desorption (NH_3_-TPD). The toluene adsorption performances of the two zeolite systems were tested with toluene as the target pollutant. The adsorption mechanism were investigated using adsorption kinetics and acid site analysis, and the performances of the two zeolites were compared. This study aims to develop low-cost and efficient VOC adsorbents and provide a theoretical basis for the resource utilization of diatomite.

## 2. Experimental

### 2.1. Materials

Sodium hydroxide (flakes, AR, 96%), kieselguhr, hydrochloric acid (AR, 36.0~38.0%) and toluene (AR, 99.5%) were purchased from China National Pharmaceutical Group Corporation (CNPGC, Beijing, China). Sodium aluminate (AR) and the commercial ZSM-5 zeolite (SiO_2_/Al_2_O_3_ ≈ 38~40) were purchased from Shanghai Macklin Biochemical Co., Ltd. (Shanghai, China).

### 2.2. Characterization

The crystalline structures of the adsorbents were characterized using an X-ray diffractometer (XRD, Rigaku SmartLab SE, Rigaku Corporation, Tokyo, Japan) with Cu Kα radiation. The XRD instrument was operated at 40 kV and 40 mA in the 2θ scan range from 5° to 50° with a step size of 0.02°. Scanning electron microscopy (SEM) was performed using a GeminiSEM 300 electron microscope (Carl Zeiss AG, Oberkochen, Germany) at an accelerating voltage of 5 kV. N_2_ adsorption–desorption measurements were carried out at −196 °C using a BELSORP-Max physisorption analyzer (MicrotracBEL Corp., Osaka, Japan). The specific surface area (*S_BET_*) was calculated using the Brunauer–Emmett–Teller (BET) method. The total pore volume was determined from the nitrogen adsorption amount at a relative pressure close to unity (*P*/*P*_0_ ≈ 0.99). Fourier transform infrared spectroscopy (FTIR) was performed on a PerkinElmer FTIR spectrometer (PerkinElmer Inc., Waltham, MA, USA). The elemental composition of the samples were determined by X-ray fluorescence spectroscopy (XRF) using a Panalytical Zetium instrument (Malvern Panalytical Ltd., Malvern, UK). NH_3_ temperature-programmed (NH_3_-TPD) desorption was tested on a Micromeritics AutoChem II 2920 instrument (Micromeritics Instrument Corporation, Norcross, GA, USA). Thermogravimetric (TG) curves were tested on a Netzsch STA 449 F5 instrument (NETZSCH-Geratebau GmbH, Selb, Germany).

### 2.3. Zeolite Synthesis

**Scheme 1:**
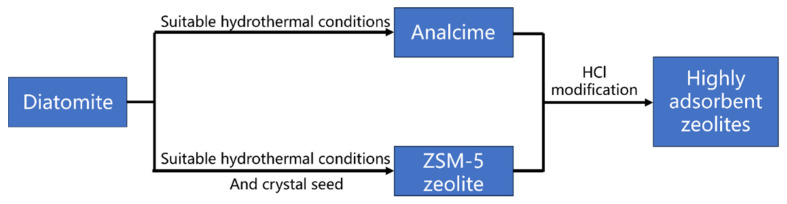
Synthesis process of analcime and the ZSM-5 zeolite.

The synthesis processes of analcime and the ZSM-5 zeolite are shown in Scheme 1. The purchased diatomite mainly consisted of crystalline SiO_2_ containing Si, Al, and Na (Figure S1). The hydrothermal conditions for analcime synthesis were optimized by adjusting the crystallization temperature (150–190 °C), time (12–36 h), and material ratio, and the optimal conditions were determined as follows: diatomite, NaOH, NaAlO_2_, and H_2_O were put into a 100 mL polytetrafluoroethylene liner (37 mL of water was used) in the ratio of SiO_2_:Al_2_O_3_:Na_2_O:H_2_O = 30:1:14:516. After crystallization at 170 °C for 24 h, the filtrate was washed with deionized water to pH = 7, and finally dried at 60 °C to obtain analcime samples (Figure S5, Table S1).

Diatomite was pretreated to obtain an amorphous silica powder containing Si, O, Al, and Na, as shown in Figures S2 and S3. The pretreatment process is shown in Figure S4. For ZSM-5 zeolite synthesis, the optimal crystallization conditions were determined by optimization experiments (Figure S6, Table S2): the raw materials were put into a 100 mL PTFE liner (16 mL water was used) in the ratio of SiO_2_:Al_2_O_3_:Na_2_O:H_2_O = 38:1:3.5:759. The commercial ZSM-5 zeolite, corresponding to 10 wt% of SiO_2_, was added as a seed to promote crystallization [29,31]. After crystallization at 180 °C for 36 h, the filtrate was washed with deionized water to pH = 7 and finally dried at 60 °C to obtain ZSM-5 zeolite samples.

To ensure reproducibility, the detailed reagent dosages, alkalinity, crystallization temperature, and crystallization time used for the synthesis of analcime and the ZSM-5 zeolite are provided in Tables S1 and S2. After hydrothermal crystallization and solid products crystallization, the solid products were separated by centrifugation, washed several times with deionized water until the filtrate was nearly neutral, and then dried at 60 °C overnight. Each synthesis experiment was repeated at least three times under the same conditions, and no obvious differences in crystal phase or morphology were observed.

### 2.4. Zeolite Modification

Hydrochloric acid solutions of 0.1, 0.25, 0.5, and 1.0 mol·L^−1^ were prepared for zeolite modification. The concentration of hydrochloric acid was selected based on previous studies [32,33] and pre-experiments, where low concentrations (≤0.25 mol·L^−1^) had little effect on pore structure, and high concentrations (>1.0 mol·L^−1^) caused severe damage to the zeolite framework. A total of 3 g of zeolite sample was weighed into a 100 mL conical flask, and 60 mL of hydrochloric acid with corresponding concentration was added according to the solid–liquid ratio of 1:20. The samples were stirred at 800 rpm for 3 h, then dried at 60 °C, and collected to obtain acid-modified zeolite powders. For acid modification, the same solid-to-liquid ratio was used for all samples to ensure comparability. After treatment, the samples were washed with deionized water until the filtrate was nearly neutral, dried at 60 °C, and collected as acid-modified zeolite powders. Each acid modification experiment was repeated at least three times, and the obtained samples showed good reproducibility in texture properties and adsorption performance. The unmodified analcime sample was named F-0, and the analcime samples modified with different concentrations of hydrochloric acid were named as F-0.1, F-0.25, F-0.5, and F-1.0 in turn. Similarly, the unmodified ZSM-5 zeolite and modified samples were named Z-0, Z-0.1, Z-0.25, Z-0.5, and Z-1.0.

### 2.5. Dynamic Adsorption Tests

The adsorption properties of the F-series and Z-series samples were measured using a fixed-bed tube (500 mm length, 8 mm inner diameter) combined with an online Nexis GC-2030 AF gas chromatograph (GC) equipped with a flame ionization detector (FID) to monitor the outlet concentration of toluene. Before testing, 1 g of each sample was treated at 60 °C for 24 h to remove volatile impurities, then loaded into a quartz tube for toluene adsorption experiments. The toluene concentration was set to 400 ppm for F-series samples and 600 ppm for Z-series samples because Z-series zeolites have better adsorption performance, and a higher concentration could better reflect the difference in adsorption capacity between the modified and unmodified samples. The total gas flow rate was 400 mL·min^−1^, and the adsorption temperature was set to 40 °C. The adsorption capacity *q_e_* (mg·g^−1^) was calculated by integrating the adsorption curve using Formula (1).

(1) $$q_{e}=\frac{F\times C_{0}\times 10^{-6}}{m}\times (t_{s}-\int_{0}^{t_{s}}\frac{C_{t}}{C_{0}}dt)$$

where *F* (mL·min^−1^) is the gas flow rate; *C*_0_ (mg·m^−3^) and *C_t_* (mg·m^−3^) represent the inlet and outlet gas concentrations, respectively; *m* (g) is the mass of the adsorbent; *t_s_* (min) is the saturation time, which is defined as the time taken for the outlet concentration to reach 95% of C_0_ for the first time.

All dynamic adsorption tests were performed at least three times under identical conditions. The average values were used to calculate the adsorption capacity, and the relative deviations among repeated tests were within an acceptable range. Before each test, the adsorbent was pretreated under the same conditions to remove volatile impurities and ensure comparable initial states.

## 3. Results and Discussion

### 3.1. Characteristics of Adsorbent

**Figure 1:**
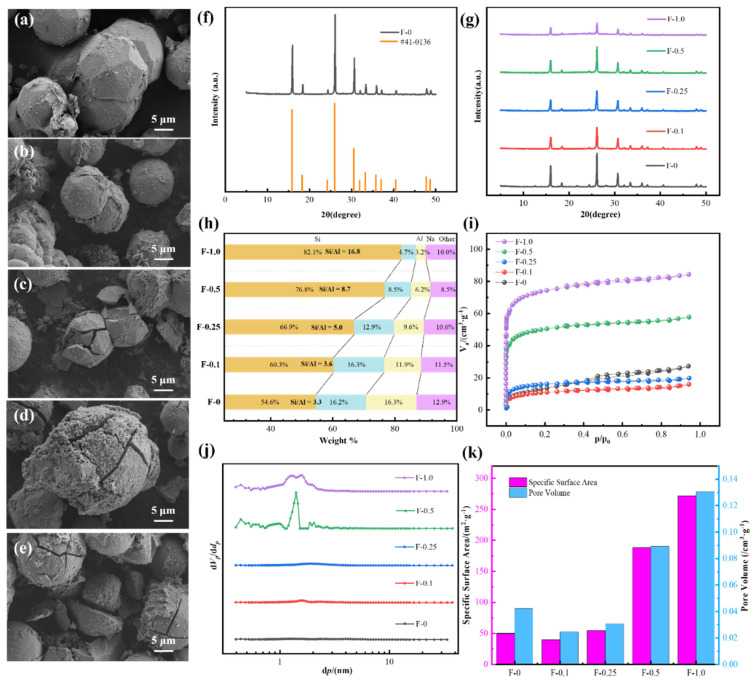
Characterization of F-series samples. (**a**–**e**) SEM images of F-0, F-0.1, F-0.25, F-0.5, and F-1.0, respectively. (**f**,**g**) XRD patterns of F-series samples compared with standard reference patterns. (**h**) XRF results of F-series samples. (**i**) N_2_ adsorption–desorption isotherms (detailed Y-axis unit: cm^3^ g^−1^ STP). (**j**) Pore size distribution. (**k**) Comparison of BET specific surface area and pore volume.

The optimization of hydrothermal conditions for analcime synthesis is shown in Figure S5 and Table S1. SEM images of the F-series samples are shown in Figure 1. The analcime sample showed a clear icositetrahedral morphology, which is a typical feature of analcime (Figure 1a) [34,35]. The irregularities scattered around the crystals were presumed to be diatomite not completely destroyed by NaOH. Obvious cracks were observed on the surface of the HCl-treated analcime, and they became denser as the HCl concentration increased (Figure 1b–e). This is due to the high Al content of the framework of the F-series samples, and HCl caused greater damage to the framework structure.

The XRD spectrum of the analcime particles is shown in Figure 1f, showing the characteristic diffraction peaks of analcime. Figure 1g shows the XRD spectrum of the F-series samples. Compared with F-0, the acid-treated samples showed weaker characteristic diffraction peaks, with the most pronounced decrease at F-1.0. This is due to the acid attacking the aluminum atoms of the zeolite framework, causing defects in the zeolite crystals and thus a decrease in crystallinity [33]. The XRF (Figure 1h) results show that the higher the concentration of acid treatment, the greater the Si/Al ratio of the resulting samples, again due to acid attacking the aluminum in the zeolite framework.

The N_2_ adsorption–desorption isotherms (Figure 1i) showed that all F-series samples exhibited type I isotherms, indicating that the adsorbents are mainly microporous. Figure 1j demonstrates the pore size distribution of the F-series samples. The acid treatment was capable of forming some pores with larger pore sizes, especially for the two samples F-0.5 and F-1.0, both of which showed significant size distributions at 1.3~1.6 nm, but still belong to the category of micropores. As shown in Table 1 and Figure 1k, treatment with low HCl concentrations, namely 0.1 and 0.25 mol·L^−1^, resulted in a slight decrease in specific surface area compared with F-0. This may be caused by the de-alumination effect of HCl on the framework disintegrating the smaller analcime particles. The samples treated with higher concentrations of HCl (F-0.5, F-1.0) exhibited larger specific surface areas. However, the increased BET surface area did not translate into a proportional increase in toluene uptake, indicating that adsorption was also governed by pore accessibility, diffusion resistance, pore matching, and available adsorption sites.

**Figure 2:**
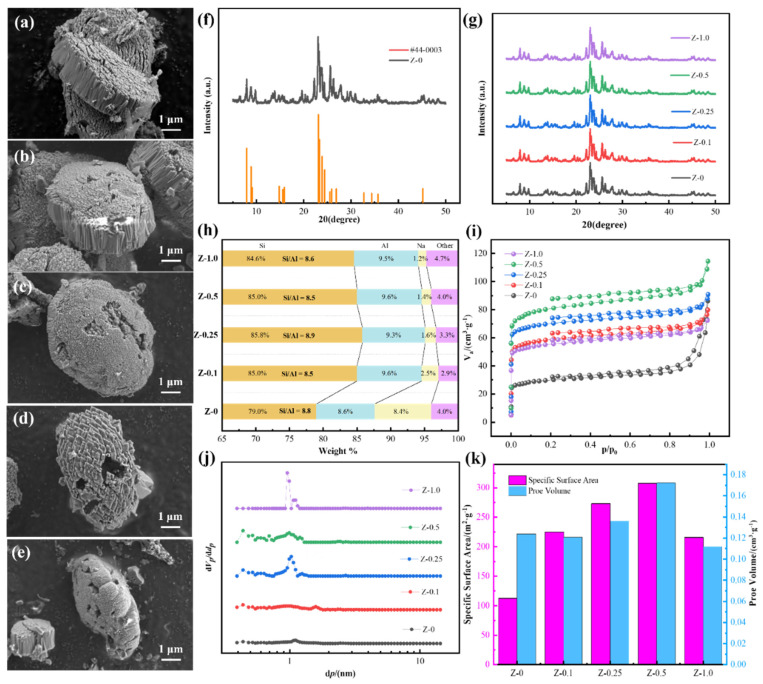
Characterization of Z-series samples. (**a**–**e**) SEM images of Z-0, Z-0.1, Z-0.25, Z-0.5, and Z-1.0, respectively. (**f**,**g**) XRD patterns of Z-series samples compared with standard reference patterns. (**h**) XRF results of Z-series samples. (**i**) N_2_ adsorption–desorption isotherms (detailed Y-axis unit: cm^3^ g^−1^ STP). (**j**) Pore size distribution. (**k**) Comparison of BET specific surface area and pore volume.

The optimization of hydrothermal conditions for ZSM-5 zeolite synthesis is shown in Figure S6 and Table S2. SEM images of the Z-series samples are shown in Figure 2. The Z-0 sample showed an overall hexagonal crystal shape (Figure 2a), which is typical of ZSM-5 zeolites [36]. However, the crystal boundaries showed filamentous structures similar to those of mordenite [37]. The irregular material scattered around is presumed to be hydrothermal quartz that has not fully crystallized [38]. Obvious pore structures were observed on the surface of the HCl-treated ZSM-5 zeolite, and the pores became denser with increasing HCl concentration (Figure 2b–e). Different from the F-series samples, the Z-series zeolite crystals did not show overall cracking, which is due to the lower Al content of the Z-series zeolite framework, and the lesser extent of damage to the framework structure by HCl.

The XRD patterns of the Z-series samples are shown in Figure 2f. The characteristic diffraction peaks matched those of standard ZSM-5 zeolite. In addition to the characteristic peaks of the ZSM-5 zeolite, a small number of characteristic peaks of MOR zeolite (near 26.5°) were present, which is consistent with the SEM images. This may be due to the fact that the synthesis conditions of the ZSM-5 zeolite and MOR zeolite are extremely similar, and thus a small amount of MOR zeolite was formed during the crystallization of ZSM-5 zeolite. Figure 2g shows the XRD of the acid-modified ZSM-5 zeolite sample, where the characteristic peaks of the acid-treated sample showed almost no decrease in peak size compared to Z-0. The Z-series zeolites contained less framework aluminum; therefore, acid treatment did not cause significant structural damage. This was verified by the XRF results (Figure 2h), which showed that the silica–aluminum ratios of the resulting samples did not change significantly as the concentration of the acid treatment was increased.

The N_2_ adsorption–desorption isotherms (Figure 2i) showed that the adsorption–desorption isotherms for all Z-series samples were relatively steeper in the low-pressure region (*p*/*p*_0_ = 0–0.1) and in the high-pressure region (*p*/*p*_0_ = 0.9–1.0), and showed a type-II isotherm, suggesting that micropores were present in the adsorbent. Some non-porous structures were also present, which may be due to the small amount of hydrothermal quartz in the sample. Figure 2j shows the pore size distributions of the Z-series samples, in which more obvious pore size distributions could be observed around 1.0 nm for the three samples, Z-0.25, Z-0.5 and Z-1.0, suggesting that the pore-forming effect of the acid modification is positively correlated with the concentration of the acid treatment. From Figure 2k and Table 2, it can be seen that the hydrochloric acid treatment increased the specific surface area and pore volume of the zeolites to different degrees compared with that of the original samples, which was also positively correlated with the concentration of the acid treatment. However, Z-1.0 is an exception, which may be due to the de-alumination of the skeleton by the high concentration of hydrochloric acid and the disintegration of some zeolite particles. For the Z-series samples, the marked increase in BET surface area led to only limited adsorption enhancement, because some newly formed narrow micropores may be inaccessible to toluene, while the ZSM-5 channels impose additional diffusion constraints.

Unlike the F-series samples, the Z-series zolite crystals did not show obvious overall cracking after acid modification. In addition to the lower framework Al content, the ring structure of ZSM-5 zeolite has higher structural stability than that of analcime, making it more resistant to acid corrosion [36,38]. The N_2_ adsorption–desorption isotherms of F-series samples showed a typical type I isotherm without an obvious hysteresis loop, indicating a pure microporous structure. For Z-series samples, a weak hysteresis loop was observed in the high-pressure region (*p*/*p*_0_ = 0.9–1.0), which was attributed to the presence of a small amount of mesopores formed by acid modification [39]. Thus, the adsorption performance of acid-modified ZSM-5 is limited mainly by pore accessibility and diffusion rather than by the total BET surface area.

### 3.2. Adsorption Performance

**Figure 3:**
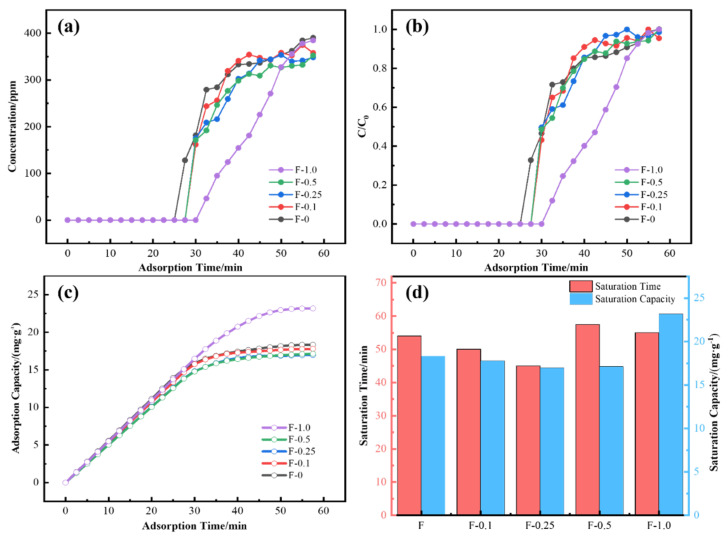
Toluene adsorption performance of F-series samples: (**a**) outlet concentration as a function of time, (**b**) breakthrough curve, (**c**) cumulative adsorption capacity curves, and (**d**) comparison of saturation time and saturation adsorption.

Toluene was used as the target pollutant to evaluate the adsorption performance of the F-series samples. At the beginning of the adsorption penetration, the outlet concentration changed more steeply with time and then leveled off (Figure 3a,b). The adsorption capacities of F-0.1, F-0.25, and F-0.5 were close to that of F-0, whereas the adsorption amount of F-1.0 showed a significantly higher adsorption capacity than the other four samples (23.2 mg·g^−1^, more than 26.6% of the amount adsorbed by F-0) (Figure 3c). Details are given in Table S3. Toluene has a molecular diameter of 0.68 nm [40,41], which is similar to the intracrystalline pore size of the F-series samples, and intracrystalline micropores can provide the main adsorption sites [32]. The specific surface areas of F-0, F-0.1, and F-0.25 were similar; therefore, their saturation times were also close (Figure 3d). The specific surface areas of F-0.5 and F-1.0 were higher, and the presence of more pore channels promoted the diffusion of toluene within the adsorbent [39]. Thus, the saturation time was longer and the adsorption performance was better.

**Figure 4:**
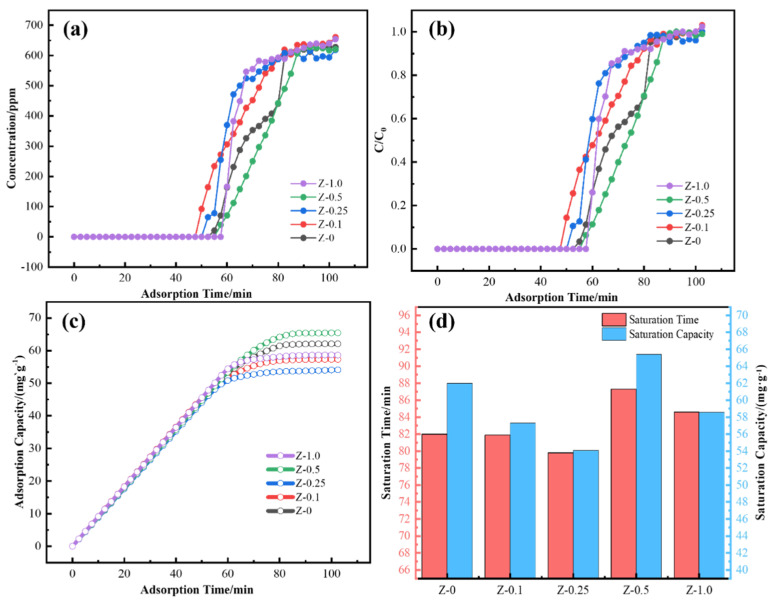
Toluene adsorption performance of Z-series samples: (**a**) outlet concentration as a function of time, (**b**) breakthrough curves, (**c**) cumulative adsorption capacity curves, and (**d**) comparison of saturation time and saturation adsorption capacity.

The toluene adsorption curves of the Z-series samples are shown in Figure 4a,b. At the initiation of penetration, the exit concentrations of Z-0, Z-0.1, Z-0.25, and Z-1.0 changed more steeply with time and then increased slowly. In contrast, Z-0.5 showed a more uniform breakthrough process. This may be due to the more uniform distribution of pores within Z-0.5. The extended adsorption time and high adsorption capacity of Z-0.5 are attributed to the large specific surface area and pore volume of the sample (Figure 4c,d). The Z-0.5 toluene adsorption capacity (65.4 mg/g) exceeded the Z-0 adsorption capacity (62.0 mg/g) by 5.5%. Combined with the N_2_ adsorption–desorption results, micropores are still dominant within the Z-series samples, and the intersection of the microporous pores of the ZSM-5 zeolite is the main adsorption site for toluene [42]. The adsorption of toluene by Z-series zeolites is generally higher than that of F-series, which is due to the higher specific surface area and pore volume of Z-series zeolites. Different inlet toluene concentrations were used for the F-series and Z-series samples because preliminary tests showed markedly different adsorption capacities. A lower inlet concentration was applied to the F-series samples to obtain reliable breakthrough curves, whereas a higher concentration was used for the Z-series samples to avoid excessively long breakthrough times. Therefore, a comparison within each series is reliable under identical test conditions, while direct quantitative comparison between the two series samples should be interpreted with caution; nevertheless, the Z-series samples showed much higher toluene adsorption capacity owing to its larger specific surface area, higher pore volume, and more accessible microporous channels.

For both the F- and Z-series samples, the Bangham model provided a better fit (R^2^ = 0.95–0.98 for F-series; R^2^ = 0.92–0.97 for Z-series) than the pseudo-first-order model (R^2^ = 0.76–0.95 and 0.73–0.94, respectively), as shown in Tables S5 and S6. The fact that the exponent z exceeded 1 in the Bangham model further indicates that pore diffusion is likely the rate-limiting step in the adsorption process. In terms of adsorbent performance, the ZSM-5 zeolite synthesized in this study without OSDA exhibited key properties comparable to those of OSDA-synthesized references (Table 3). Compared with the OSDA-synthesized ZSM-5 zeolites listed in Table 3, the acid-modified ZSM-5 in this study had a slightly lower specific surface area but a comparable toluene adsorption capacity (65.4 mg·g^−1^ vs. 58.2–81 mg·g^−1^), and its synthesis process was more environmentally friendly and low-cost without OSDA. In contrast, analcime-based zeolites in this study demonstrated relatively inferior adsorption performance. Regeneration and cyclic adsorption tests are still needed to further assess the long-term stability and practical applicability of these adsorbents. Further regeneration, cyclic adsorption, and humidity-resistance tests are needed to assess the long-term stability and practical applicability of these adsorbents under realistic VOC conditions.

### 3.3. Adsorption Mechanism

**Figure 5:**
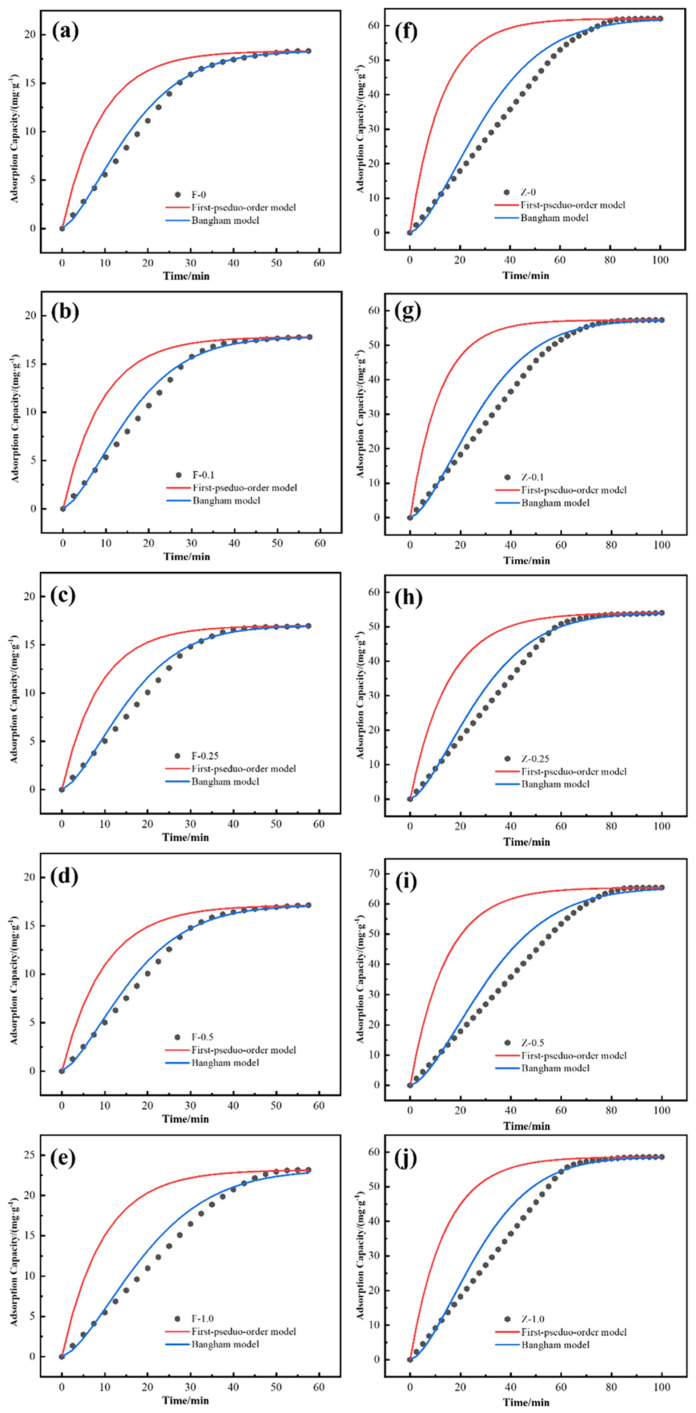
Fitting results of the pseudo-first-order model and Bangham model for toluene adsorption: (**a**–**e**) F-series samples, including F-0, F-0.1, F-0.25, F-0.5, and F-1.0; (**f**–**j**) Z-series samples, including Z-0, Z-0.1, Z-0.25, Z-0.5, and Z-1.0. The red and blue lines represent the pseudo-first-order and Bangham model fittings, respectively.

The adsorption process was analyzed using the pseudo-first-order model and the Bangham model (Figure 5 and Tables S5 and S6). The pseudo-first-order model was selected to evaluate the contribution of physical adsorption controlled by adsorbate–adsorbent interactions, whereas the Bangham model was used to describe pore-diffusion-controlled adsorption in porous materials. Therefore, the combination of these two models can provide insight into both surface adsorption and intraparticle diffusion during toluene uptake. The quasi-first-order model showed relatively high R^2^ values, indicating that physical adsorption played an important role in the adsorption process. In addition, the Bangham model also showed high R^2^ (>0.95) values, indicating that pore diffusion was important during toluene adsorption. We also analyzed the diffusion rates of the samples (Tables S7 and S8). The diffusion coefficients of the F-series samples were 23.22 × 10^−4^, 23.79 × 10^−4^, 23.99 × 10^−4^, 23.66 × 10^−4^, and 23.98 × 10^−4^ min^−1^. The diffusion coefficients of the Z-series samples were 14.96 × 10^−4^, 14.79 × 10^−4^, 14.82 × 10^−4^, 14.93 × 10^−4^, and 15.15 ×10^−4^ min^−1^. The diffusion coefficient of the Z-series samples was lower than that of the F-series samples, but the adsorption amount was higher than that of the F-series samples. These results indicate that the adsorption capacity was not only related to the diffusion rate, but was also affected by the inlet concentration (C_0_) and the number of internal adsorption sites.

**Figure 6:**
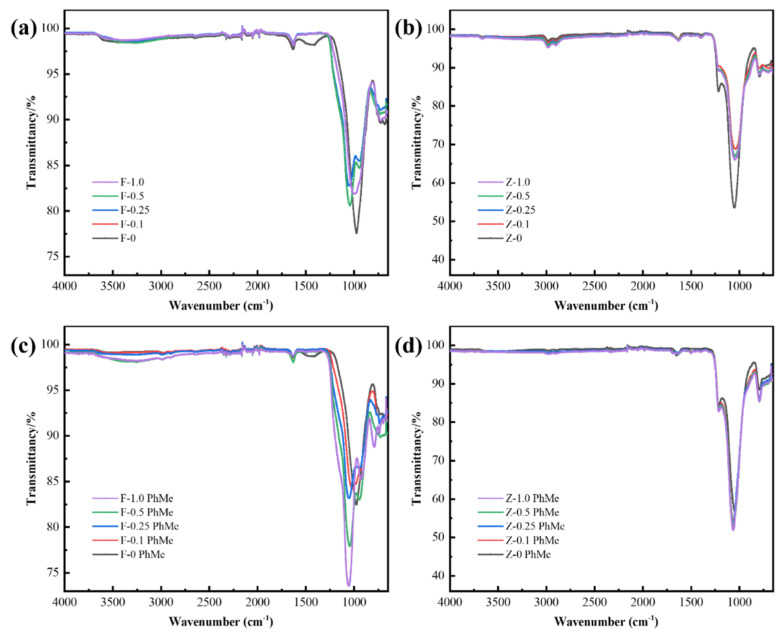
FTIR spectra of zeolite samples before and after toluene adsorption: (**a**) F-series samples before adsorption, (**b**) Z-series samples before adsorption, (**c**) F-series samples after adsorption, and (**d**) Z-series samples after adsorption. PhMe denotes toluene.

As shown in Figure 6a,b, the band at around 1020 cm^−1^ was assigned to the asymmetric stretching vibration of Si-O bonds, which is a typical characteristic peak of zeolite frameworks [46,47,48]. The de-aluminum treatment led to a shift toward higher wavelengths in the absorption band of the structurally sensitive Si-O bond asymmetric stretching vibration (1010–1160 cm^−1^) [49,50]. This was particularly evident in the F-0.5 and F-1.0 samples (Figure 6a). Based on Figure 6c,d, the higher the acid concentration used for modification, the more obvious the increase in peak intensity of the absorption peak at 1020 cm^−1^ for the samples after the adsorption of toluene. This enhancement may be associated with the overlap between the Si-O vibration of the zeolite framework and possible C-O-related vibrations of oxygen-containing aromatic species formed during toluene adsorption. In addition to the enhancement of the 1020 cm^−1^ peak (overlap of Si-O bond and C-O bond of benzyl alcohol), a weak absorption peak at 1250 cm^−1^ was observed in the F-1.0 and Z-0.5 samples after toluene adsorption, which may be related to C-O stretching vibration. However, this signal alone is insufficient to conclusively confirm the formation of benzyl alcohol or the occurrence of a carbonylation reaction [51]. In conjunction with the NH_3_-TPD results (Figure S7), the acid-modified zeolite samples (F-1.0 and Z-0.5) had a higher number of acid sites and stronger strong acid sites. Enhanced acidity within the zeolite may strengthen the interaction between toluene molecules and acidic sites and could promote the formation of oxygen-containing aromatic intermediates under certain conditions [51,52,53]. Therefore, based on the FTIR and NH_3_-TPD results, a possible contribution from specific adsorption or chemisorption is proposed, although further direct evidence, such as in situ spectroscopy or product analysis, would be required to confirm the formation of benzyl alcohol.

These results suggest that in zeolite samples with a higher degree of acid modification, a small fraction of toluene molecules may interact with acid sites or possibly form oxygen-containing aromatic species, which could contribute to chemisorption. The C-O bond contained in benzyl alcohol presented an absorption peak around 1000 cm^−1^ on FTIR, which highly overlapped with the asymmetric stretching absorption peak of the zeolite’s own Si-O bond. Thus, the absorption band at around 1020 cm^−1^ became stronger after toluene adsorption for F-1.0 and Z-0.5. However, due to the overlap with the zeolite framework vibration, this band should be interpreted as indirect evidence of possible surface interactions rather than definitive proof of benzyl alcohol formation. The proportion of chemisorption in the Z-series samples may be lower than that in the F-series samples, possibly because the Z-series zeolites contained fewer acid sites (Figure S7), leading to weaker toluene–acid site interactions and a lower probability of forming oxygen-containing aromatic species [33].

**Figure 7:**
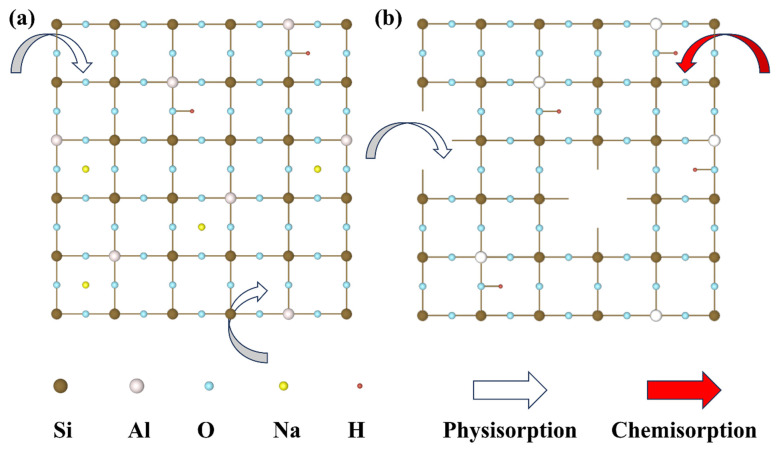
Proposed adsorption mechanisms of acid-modified zeolites: (**a**) unmodified zeolites, where physical adsorption dominates; and (**b**) acid-modified zeolites, where physical adsorption and possible chemisorption coexist.

Based on the above results, a possible adsorption mechanism of toluene on an acid-modified zeolite was presumed. As shown in Figure 7a, the number of acid sites in the unmodified zeolite samples was very rare, and there was almost no chemisorption. Therefore, toluene adsorption was mainly dominated by physical adsorption. Toluene molecules were adsorbed into the zeolite pores and cavities by pore diffusion. After acid modification (Figure 7b), the specific surface area and pore volume were increased to further improve the adsorption capacity of toluene. Meanwhile, the increased number of acid sites may enhance the interaction between toluene and the zeolite surface, resulting in a possible contribution from chemisorption. In summary, with increasing acid modification, toluene adsorption on the zeolite samples may gradually shift from predominantly physical adsorption to the combined process involving physical adsorption and possible chemisorption. This interpretation should be regarded as a plausible mechanism rather than a confirmed conclusion, because direct evidence for benzyl alcohol formation is still lacking.

## 4. Conclusions

In this study, natural diatomite was successfully converted into analcime and the ZSM-5 zeolite under OSDA-free conditions, providing a low-effective and environmentally friendly route for preparing zeolite-based VOC adsorbents from abundant mineral resources. Analcime was obtained by regulating the hydrothermal synthesis conditions, while ZSM-5 was synthesized through the combined control of hydrothermal conditions and seed induction. This strategy not only reduces the dependence on expensive organic templates, but also offers a feasible pathway for the high-value utilization of natural diatomite. Hydrochloric acid modification effectively improved the textural properties and adsorption performance of the synthesized zeolites by increasing the specific surface area, optimizing the pore structure, and generating additional adsorption sites. The optimal F-series samples, F-1.0, exhibited a specific surface area of 271.7 m^2^·g^−1^, a pore volume of 0.130 cm^3^·g^−1^, and a tolune adsorption capacity of 23.2 mg·g^−1^, which was 26.6% higher than that of the unmodified F-0. For the Z-series samples, Z-0.5 showed the best performance, with a specific surface area of 307.9 m^2^·g^−1^, a pore volume of 0.172 cm^3^·g^−1^, and a toluene adsorption capacity of 65.4 mg·g^−1^, representing a 5.5% improvement compared with unmodified Z-0. The adsorption behavior was mainly governed by physical adsorption (R^2^ > 0.95) and pore diffusion, while acid modification introduced additional acid sites that contributed to secondary chemisorption interactions. Compared with the F-series samples, the Z-series zeolites possessed larger surface area, higher pore volumes, and more accessible adsorption sites, leading to superior toluene adsorption performance. These findings highlight the importance of tailoring pore structure and surface acidity to enhance VOC adsorption. Overall, this work demonstrates that diatomite-derived, acid-modified zeolites are promising adsorbents for VOC removal. The proposed OSDA-free synthesis and simple acid modification approach provide both scientific insight into the structure–performance relationships and practical implications for developing low-cost, mineral-based adsorbents for air pollution control.

## Figures and Tables

**Table 1:** Specific surface area and pore volume of F-series samples.

Sample	Specific Surface Area/(m^2^·g^−1^)	Pore Volume/(cm^3^·g^−1^)
F-0	50.1	0.042
F-0.1	39.9	0.025
F-0.25	54.3	0.031
F-0.5	188.2	0.089
F-1.0	271.7	0.130

**Table 2:** Specific surface area and pore volume of the Z-series samples.

Sample	Specific Surface Area/(m^2^·g^−1^)	Pore Volume/(cm^3^·g^−1^)
Z-0	112.5	0.124
Z-0.1	225.1	0.121
Z-0.25	273.2	0.136
Z-0.5	307.9	0.172
Z-1.0	216.0	0.112

**Table 3:** Adsorption capacity of some zeolites synthesized using OSDA for toluene.

Zeolite Adsorbent	Raw Material ^a^	OSDA ^b^	Specific Surface Area/(m^2^·g^−1^) ^c^	Pore Volume/(cm^3^·g^−1^)	Adsorption Capacity/(mg·g^−1^)	Refs.
ZSM-5	TEOS	TPAOH/EtOH	392	0.18	81	[40]
ZSM-5/SBA-15	TEOS	TPABr, CTAB	781	1.13	100	[42]
ZSM-5	TEOS	TPABr, CTAB	418	0.33	122.9	[42]
ZSM-5	TEOS	TPAOH	308	0.22	58.2	[43]
ZSM-5	CGCS	*n*-Butylamine	344.3	0.20	81	[44]
ZSM-5	CFA	TPABr	358.5	0.24	71.0	[45]
Acid-modified analcime	Diatomite	-	271.7	0.13	23.1	This work
ZSM-5	Diatomite	-	112.5	0.124	62.0	This work
Acid-modified ZSM-5	Diatomite	-	307.9	0.172	65.4	This work

^a^ TEOS: tetraethyl orthosilicate, CGCS: coal gasification coarse slag, CFA: coal fly ash; ^b^ TPAOH: tetrapropylammonium hydroxide, EtOH: ethyl alcohol, TPABr: tetrapropylammonium bromide, CTAB: cetyltrimethylammonium bromide; ^c^ All measured by the BET method, N_2_ medium.

## Data Availability

The original contributions presented in this study are included in the article/Supplementary Materials. Further inquiries can be directed to the corresponding authors.

## Supplementary Materials

The following supporting information can be downloaded at: https://www.mdpi.com/article/10.3390/nano16140863/s1, Figure S1: Diatomite XRF test results.; Figure S2: XRD test results of diatomite standard card, diatomite, and activated diatomite. The activated diatomite was verified to exhibit amorphous silica; Figure S3: XRF results of amorphous silica powder; Figure S4: Process flow diagram for the preparation of amorphous silica from diatomite. Figure S5: XRD spectrum of analcime samples (F1~F6) synthesised under different conditions; Figure S6: XRD spectrum of ZSM-5 zeolite samples (Z1~Z5 and Commercial ZSM-5 zeolite noted as Z-com) synthesised under different conditions; Figure S7: (a) NH3-TPD test results for F-0 and F-1.0. (b) NH3-TPD test results for Z-0 and Z-0.5; Table S1: Synthesis conditions and relative crystallinity of samples F1 to F6 (F5 was used as the standard sample, and three characteristic peaks at 15.8°, 25.9° and 30.5° were selected from the standard card); Table S2: Synthesis conditions and relative crystallinity of samples Z1 to Z5 (Z-com was used as the standard sample, and three characteristic peaks at 7.94°, 8.84°, 23.08°, 23.9°, and 24.4° were selected from the standard card); Table S3: Saturation capacity and saturation time of F-series samples; Table S4: Saturation capacity and saturation time of Z-series samples; Table S5: The fitting parameters of two kinds of dynamic equations of F-series samples; Table S6: The fitting parameters of two kinds of dynamic equations of Z-series samples; Table S7: Parameters related to the diffusion of toluene on F-series samples; Table S8: Parameters related to the diffusion of toluene on Z-series samples.
