# Supplementary material for: Synthesis of Analcime and ZSM-5 Zeolite by Diatomite Without Organic Structure-Directing Agent and Adsorption Properties of Their Acid-Modified Samples on Toluene
Source: Nanomaterials (Basel). 2026 Jul 13;16(14):863. doi: 10.3390/nano16140863 (PMC13414642; doi:10.3390/nano16140863)
Supplement: Supplementary file 1 [file nanomaterials-16-00863-s001.zip › nanomaterials-4403099-supplementary.pdf]

Supporting Information

Synthesis of Analcime and ZSM-5 Zeolite by Diatomite Without Organic Structure-Directing Agent and Adsorption Properties of Their Acid-Modified Samples on Toluene

Fanghui Pan [1,]*, Jianxiang Wang [2], Javed Iqbal [3], Fei Yu [1,4,]* and Jie Ma [1,2,5]

[1] Water Resources and Water Environment Engineering Technology Center, Xinjiang Key Laboratory of Engineering Materials and Structural Safety, School of Civil Engineering, Kashi University, Kashi 844000, China; jma@tongji.edu.cn
[2] Research Center for Environmental Functional Materials, State Key Laboratory of Water Pollution Control and Green Resource Recycling, College of Environmental Science and Engineering, Tongji University, Shanghai 200092, China; 2130553@tongji.edu.cn
[3] Department of Chemistry, College of Science, University of Bahrain, Zallaq 1054, Bahrain; javed.iqbal@uaf.edu.pk
[4] College of Oceanography and Ecological Science, Shanghai Ocean University, No 999, Huchenghuan Road, Shanghai 201306, China
[5] Shanghai Institute of Pollution Control and Ecological Security, Shanghai 200092, China
* Correspondence: pfh2024@ksu.edu.cn (F.P.); f-yu@shou.edu.cn (F.Y.)

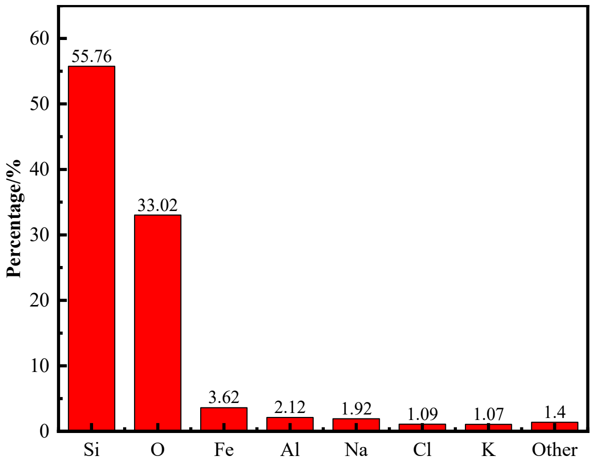

Figure S1 Diatomite XRF test results.

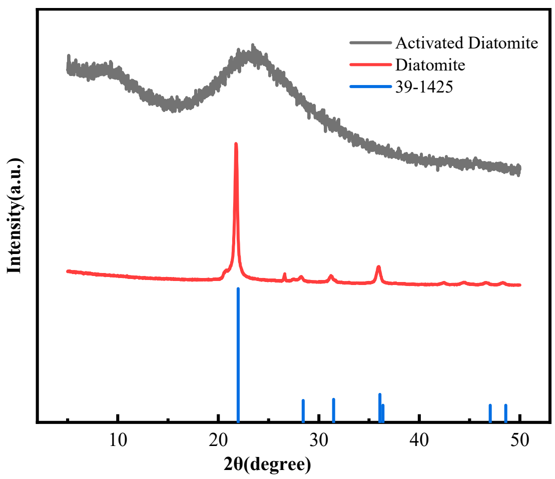

Figure S2 XRD test results of diatomite standard card, diatomite, and activated diatomite. The activated diatomite was verified to exhibit amorphous silica.

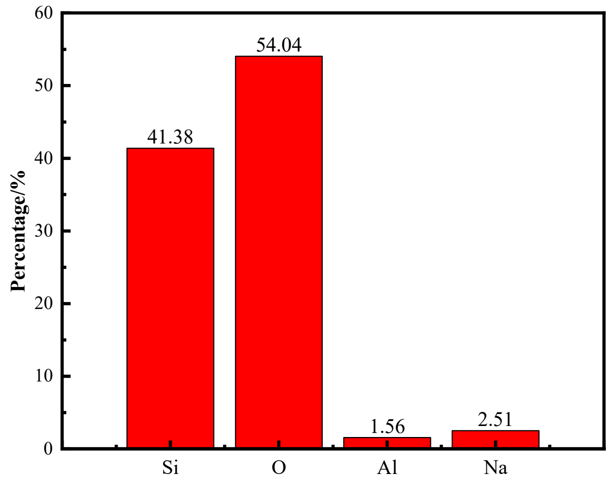

Figure S3 XRF results of amorphous silica powder.

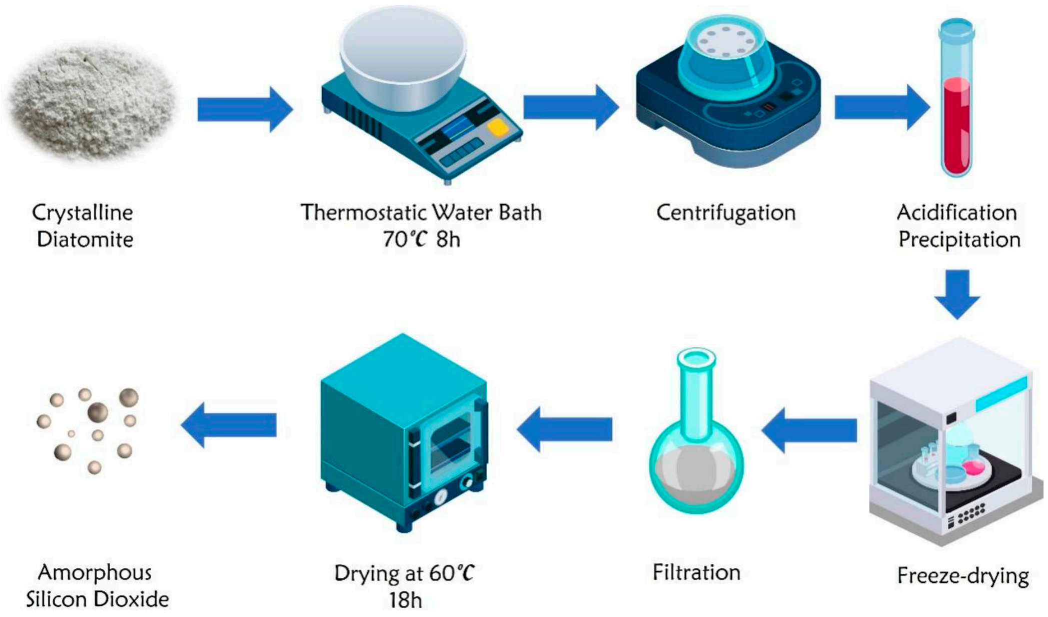

Figure S4 Process flow diagram for the preparation of amorphous silica from diatomite

$$\text{Relative crystallinity} = \frac{\sum I\ of\ characteristic\ peaks\ of\ target\ samples}{\sum I\ of\ characteristic\ peaks\ of\ standard\ samples}$$

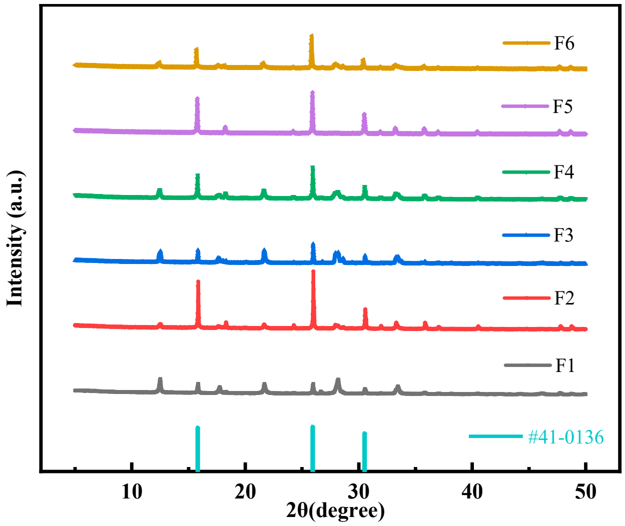

Figure S5 XRD spectrum of analcime samples (F1~F6) synthesised under different conditions.

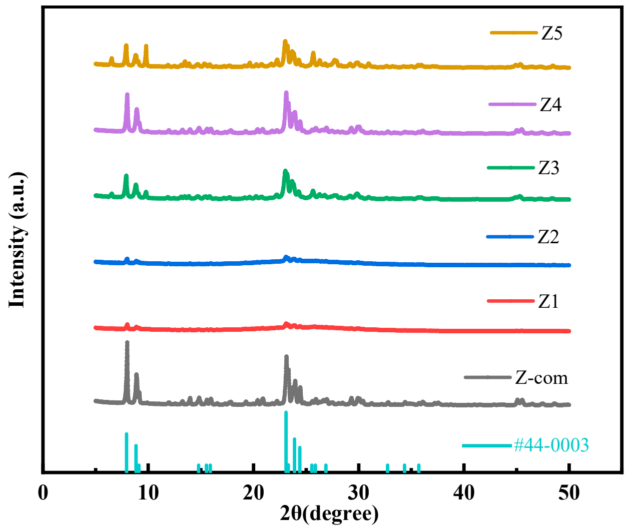

Figure S6 XRD spectrum of ZSM-5 zeolite samples (Z1~Z5 and Commercial ZSM-5 zeolite noted as Z-com) synthesised under different conditions.

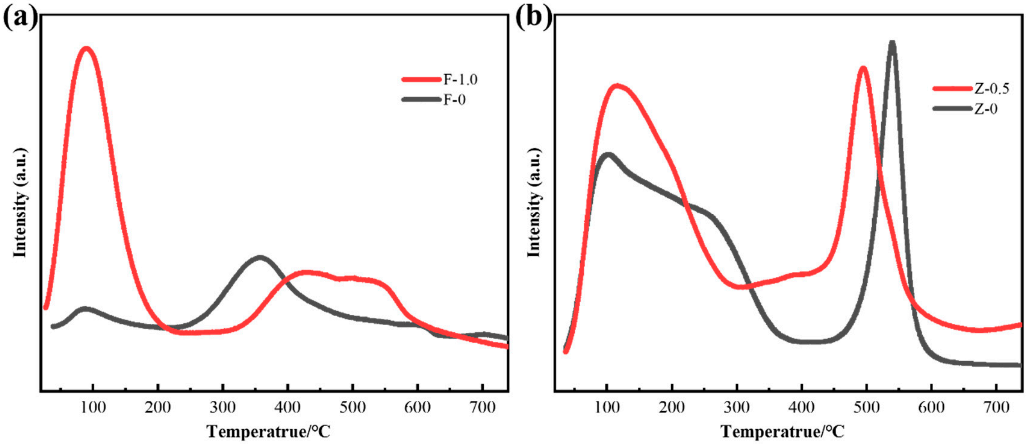

Figure S7 (a) NH_3-TPD test results for F-0 and F-1.0. (b) NH_3-TPD test results for Z-0 and Z-0.5.

The adsorption process of acetone on the samples is fitted by two kinetic models, the pseudo-first-order model and Bangham model. The equations are shown as follows.

The pseudo-first-order model:

$$q_t = q_e \times (1 - e^{-k_1 t})$$

Bangham model:

$$q_t = q_e - \frac{q_e}{e^{k_2 t^z}}$$

The diffusion coefficients:

$$\frac{q_t - q_0}{q_e - q_0} = \frac{6}{\sqrt{\pi}} \left(\frac{D}{r_0^2}\right)^{\frac{1}{2}} \sqrt{t}$$

Where q_0 (mg g^{-1}) is the toluene adsorption capacity at t=0 min; k_1, k_2 are the rate constant for pseudo-first-order model and Bangham model, respectively; the adsorption capacity of toluene at time t is q_t (mg g^{-1}); z is a constant.

Table S1. Synthesis conditions and relative crystallinity of samples F1 to F6 (F5 was used as the standard sample, and three characteristic peaks at 15.8°, 25.9° and 30.5° were selected from the standard card)

Sample number	SiO_2/Al_2O_3	Na_2O/Al_2O_3	Hydrothermal time	Relative crystallinity
**F1**	20	10	12	30.1%
**F2**	30	14	12	128.6%
**F3**	40	19	12	45.9%
**F4**	50	23	12	72%
**F5**	30	14	24	100%
**F6**	30	19	24	65.5%

Table S2. Synthesis conditions and relative crystallinity of samples Z1 to Z5 (Z-com was used as the standard sample, and three characteristic peaks at 7.94°, 8.84°, 23.08°, 23.9°, and 24.4° were selected from the standard card)

Sample number	Crystal seeds content	SiO_2/Al_2O_3	Na_2O/Al_2O_3	H_2O/SiO_2	Hydrothermal time	Relative crystallinity
**Z1**	5%	38	1.68	22	48	21.3%
**Z2**	5%	38	1.68	28	48	21.9%
**Z3**	10%	38	3.46	20	30	55.2%
**Z4**	10%	38	3.46	20	36	78.3%
**Z5**	10%	38	3.46	20	42	50.5%

Table S3. Saturation capacity and saturation time of F-series samples

Samples	Saturation capacity/($mg \cdot g^{-1}$)	Saturation time/(min)
F-0	18.3	54
F-0.1	17.8	50
F-0.25	17.0	45
F-0.5	17.1	57
F-1.0	23.2	55

Table S4. Saturation capacity and saturation time of Z-series samples

Samples	Saturation capacity/($mg \cdot g^{-1}$)	Saturation time/(min)
Z-0	62.0	82
Z-0.1	57.3	82
Z-0.25	54.1	80
Z-0.5	65.4	87
Z-1.0	58.6	85

Table S5. The fitting parameters of two kinds of dynamic equations of F-series samples

		F-0	F-0.1	F-0.25	F-0.5	F-1.0
First-pseduo-order model	k_1 (min^{-1})	0.1098	0.1094	0.1147	0.1021	0.1046
	R^2	0.869	0.947	0.943	0.938	0.763
Bangham model	k_2 (min^{-1})	0.0142	0.0134	0.0125	0.0136	0.0091
	z	1.458	1.487	1.511	1.462	1.510
	R^2	0.980	0.982	0.979	0.983	0.947

Table S6. The fitting parameters of two kinds of dynamic equations of Z-series samples

		Z-0	Z-0.1	Z-0.25	Z-0.5	Z-1.0
First-pseduo-order model	k_1 (min^{-1})	0.0783	0.0850	0.0665	0.0713	0.0845
	R^2	0.812	0.829	0.941	0.809	0.729
Bangham model	k_2 (min^{-z})	0.0040	0.0046	0.0053	0.0036	0.0041
	z	1.550	1.551	1.516	1.559	1.579
	R^2	0.943	0.955	0.965	0.923	0.950

Table S7. Parameters related to the diffusion of toluene on F-series samples

Samples	D/r_0^2 ×10^{-4} (min^{-1})	a	b	R^2
F-0	23.22	0.1631	-0.1275	0.950
F-0.1	23.79	0.1651	-0.1334	0.944
F-0.25	23.99	0.1658	-0.1394	0.946
F-0.5	23.66	0.1647	-0.1408	0.950
F-1.0	23.98	0.1658	-0.2105	0.961

Table S8. Parameters related to the diffusion of toluene on Z-series samples

Samples	$D/r_0^2 \times 10^{-4}$ (min^{-1})	a	b	R^2
Z-0	14.96	0.1309	-0.2254	0.964
Z-0.1	14.79	0.1302	-0.1900	0.960
Z-0.25	14.82	0.1303	-0.1809	0.951
Z-0.5	14.93	0.1308	-0.2411	0.965
Z-1.0	15.13	0.1317	-0.2019	0.954
